# Antimicrobial Drug Resistance, Regulation, and Research^1^

**DOI:** 10.3201/eid1202.050078

**Published:** 2006-02

**Authors:** Joshua P. Metlay, John H. Powers, Michael N. Dudley, Keryn Christiansen, Roger G. Finch, on behalf of the Second Colloquium of the International Forum on Antibiotic Resistance

**Affiliations:** * VA Medical Center, Philadelphia, Pennsylvania, USA;; † University of Pennsylvania School of Medicine, Philadelphia, Pennsylvania, USA;; ‡ US Food and Drug Administration, Rockville, Maryland, USA;; § Diversa Corporation, San Diego, California, USA;; ¶ Royal Perth Hospital, Perth, Western Australia, Australia;; # Nottingham City Hospital, Nottingham, United Kingdom;; ** University of Nottingham, Nottingham, United Kingdom

**Keywords:** Antibiotic resistance, Bacterial infections, Clinical trials, Drug approval, Drug industry, Drug resistance, microbial, Outcome assessment, Pharmacology, Pharmacokinetics

## Abstract

Innovative regulatory and legislative measures to stimulate and facilitate the development of new antimicrobial drugs are needed. We discuss research approaches that can aid regulatory decision making on the treatment of resistant infections and minimization of resistance selection. We also outline current and future measures that regulatory agencies may employ to help control resistance and promote drug development. Pharmacokinetic/pharmacodynamic research models offer promising approaches to define the determinants of resistance selection and drug doses that optimize efficacy and reduce resistance selection. Internationally, variations exist in how regulators use drug scheduling, subsidy restrictions, central directives, educational guidelines, amendments to prescribing information, and indication review. Recent consultations and collaborations between regulators, academics, and industry are welcome. Efforts to coordinate regulatory measures would benefit from greater levels of international dialogue.

## Abstract 2

Research models and regulatory measures could aid in developing antimicrobial drugs to address bacterial resistance.

Strategies for addressing antimicrobial drug resistance stress the need for new drugs (*1**–**3*), and yet the rate of drug development is in decline (Figure 1) (*4*). The Infectious Diseases Society of America (IDSA) (*5*), the World Health Organization (*6*), and other experts (*7*) have drawn attention to this potentially serious threat to public health. Possible reasons include the slow growth in antimicrobial drug sales, caused in part by guidelines for conservative and generic drug prescribing. Resistance limits the market life of antimicrobial drugs, while limited markets exist for agents only active against resistant pathogens. Developers face challenges in demonstrating that new drugs are as safe as established agents. Finally, researchers have found converting pharmacologic targets into commercially viable drugs to be difficult.

**Figure 1:**
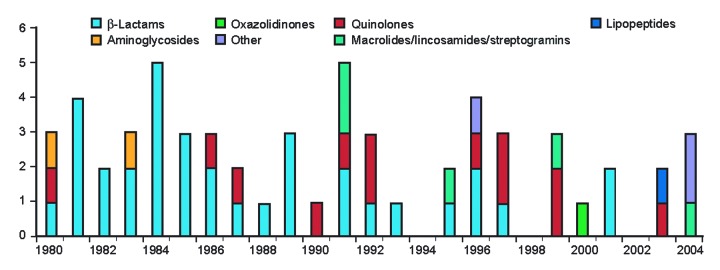
US Food and Drug Administration approvals of systemic antibacterial new molecular entities, 1980–2004. Adapted with permission from Blackwell Scientific (*4*).

Regulatory bodies have roles within collaborative responses to improve the prevention and treatment of infections caused by resistant bacteria. However, in an era of emerging drug resistance, controlled clinical data are often not available to guide regulatory policy. In the first half of this article, we discuss pharmacokinetic/pharmacodynamic (PK/PD) research approaches that can aid regulatory decision making on the treatment of resistant infections and minimization of resistance selection. In the second half, we outline measures that regulatory agencies may use to help control resistance and facilitate drug development.

## Scientific Basis for Regulatory Responses to Resistance

Measures to control resistance should be based on scientific evidence concerning its effect on human health and the effectiveness of available interventions. Unfortunately, quantitative data concerning the clinical implications of resistance are lacking for many common infections (*8*). PK/PD models may be used to identify the determinants and implications of resistance, although clinical data on symptom resolution or survival remain the standard (*9*).

**Figure 2:**
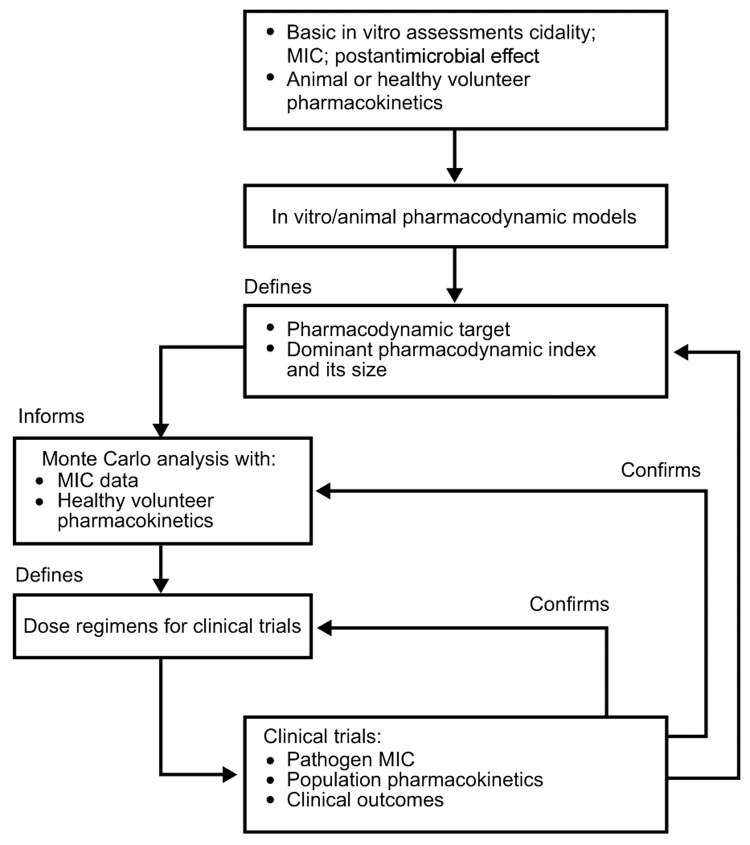
Defining the pharmacodynamic target for therapy. Adapted with permission from Blackwell Scientific (*13*).

PK/PD research aims to identify antimicrobial drug exposures relative to the in vitro MIC that best predicts efficacy and reduced selection of resistance, i.e., the PK/PD index (Figure 2) (*10**–**13*). The PK/PD index is influenced by bacterial, host, and experimental factors (*12**,**14*) but tends not to vary among strains of a bacterial species. While absolute doses (in milligrams per kilogram) associated with efficacy correlate poorly between animal models and humans, parameters of antimicrobial drug exposure relative to MIC can generate clinically relevant PK/PD indices (*11*).

### Applying PK/PD Analyses to Doses and Breakpoints

Many existing in vitro MIC susceptibility breakpoints were established both for epidemiologic surveillance and to guide therapy in individual persons. Accumulating evidence supports the use of separate breakpoints for these purposes.

**Figure 3:**
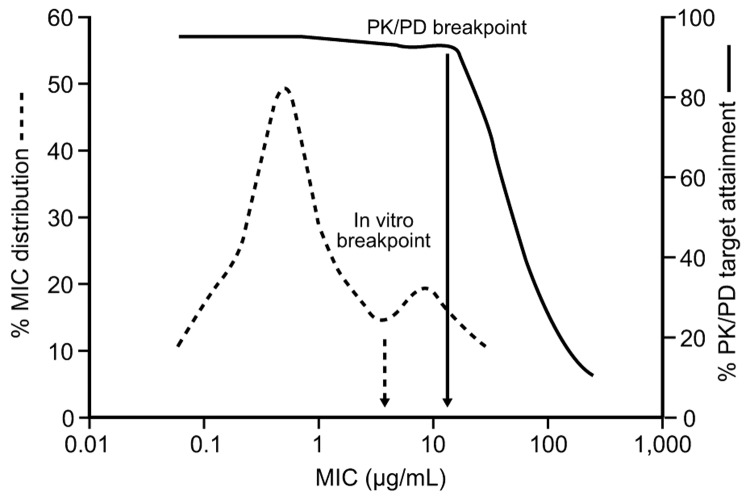
Relationship between MIC and attainment of the pharmacokinetic/pharmacodynamic (PK/PD) target for effect. Accumulating evidence supports the use of separate PK/PD breakpoints for clinical decision making, distinct from in vitro breakpoints used for epidemiologic surveillance. A breakpoint derived from PK/PD data represents the highest MIC for which the unbound plasma concentrations of the drug (after standard doses) are sufficient to achieve the target PK/PD exposure.

PK/PD data may aid the selection of clinical breakpoints. PK/PD breakpoints represent the highest MIC for which the unbound plasma concentrations of the antimicrobial drug (following standard doses) are sufficient to achieve the PK/PD target against a defined organism and for which adequate clinical data support their use (Figure 3). PK/PD targets are usually derived in vivo by using susceptible strains. The targets for strains with certain resistance mechanisms may differ. However, in several cases, studies have verified that these PK/PD targets apply in less susceptible strains (*15*).

The effects of variability within populations on attaining PK/PD targets can be probed by using Monte Carlo simulation of numerous drug exposures (*10**,**16**,**17*). The fraction of exposures that attain the PK/PD target can be determined across the MIC range of the pathogen and used to help select MIC breakpoints (*17**,**18*). The optimal dose can also be selected by analyzing PK/PD target attainment rates for fixed doses across the MIC range.

Clinical breakpoints may differ substantially from in vitro MIC breakpoints (Figure 3). In 2000, the National Committee for Clinical Laboratory Standards revised the recommended MIC breakpoints for oral β-lactams against *Streptococcus pneumoniae* in light of clinical and PK/PD data (*19*). PK/PD analyses have recently been applied to other breakpoint determinations (*10**,**16**,**19**,**20*). Controlled trials regarding the clinical relevance of discrepancies between current and proposed breakpoints are unavailable. However, case reports indicate a potential increase in treatment failures with some drug classes (and a potential failure to detect these mechanisms with reference microbiologic methods) and suggest the need for better clinical data to reassess susceptibility breakpoints for these agents.

We may also have to challenge the paradigm that interprets breakpoints as dichotomous variables associated with categoric responses such as success and failure. Reductions in susceptibility have graded effects and should instead be interpreted in terms of a reduced relative likelihood of positive outcomes.

### PK/PD Targets To Suppress Resistance

**Figure 4:**
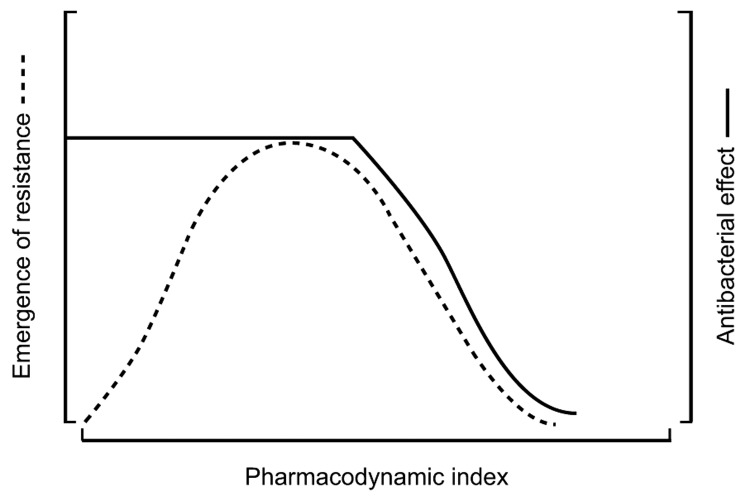
Relationship between the dominant pharmacokinetic/pharmacodynamic (PK/PD) index, efficacy, and resistance emergence in vitro (both quantified by the number of bacterial colony-forming units). The PK/PD index is related to efficacy in a sigmoid curve and the resistance emergence by an inverted U-shaped curve (*21*).

**Figure 5:**
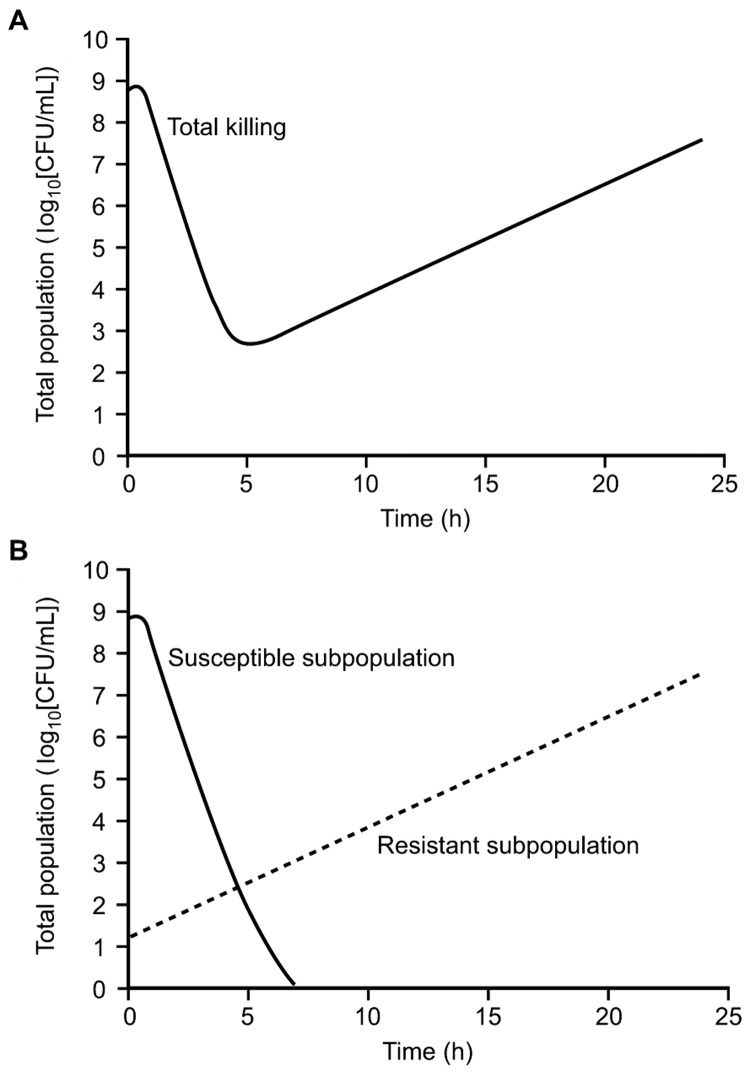
Killing pattern for a fluoroquinolone against *Pseudomonas aeruginosa* that illustrates how the initial decline and subsequent regrowth observed in the total number of colony-forming units (A) represent the sum of a decline in the susceptible subpopulation and the uninhibited growth of a resistant subpopulation (B).

Intermediate PK/PD index values may produce antibacterial effects but also select for resistant bacteria (Figure 4). This phenomenon can be conceptually described by considering an infectious bacterial inoculum as a swarm, rather than a clone. A large bacterial load is likely to contain a resistant subpopulation at baseline that is selected during antimicrobial drug therapy. This occurrence can be studied by using a mixed inoculum made up of a susceptible population and a small resistant subpopulation (Figure 5) (*16**,**18*).

Mixed-inoculum studies show that the time when the antimicrobial drug concentration exceeds the MIC is the dominant PK/PD index for the selection of penicillin-resistant *S. pneumoniae* (*22**,**23*). The ratio of the area under the concentration-time curve to the MIC (AUC/MIC) predicts fluoroquinolone resistance in this species (*21**,**24*), while the ratio of the maximum concentration and the MIC, and the AUC/MIC, predicts the selection of fluoroquinolone resistance in *Pseudomonas aeruginosa* (*16**,**25**,**26*). In each case, the PK/PD index for resistance selection is the same as that associated with microbiologic activity, although its magnitude may exceed values that can be supplied with usual or safe dosage regimens (*25**,**27*).

Jumbe et al. (*16*) calculated fluoroquinolone PK/PD targets that would amplify or suppress susceptible and resistant populations of *P. aeruginosa* in mice and prospectively validated the resulting dose regimens. These and other data (*23*) underscore the need to determine in clinical studies whether drug regimens should be directed against resistant subpopulations as well as susceptible populations. Such studies would need to correlate bacteriologic treatment failures with initial and posttreatment susceptibility data and antimicrobial drug exposure. Ultimately, they could assess the emergence of resistance among commensal flora.

### Future Directions

Although PK/PD data are increasingly valuable, detailed information on the selection and effect of resistance in patients can only be provided by studies designed for this purpose. Such studies should be disease specific and should control for the confounding effect of coexisting conditions (*28**,**29*). Outcomes research would benefit from standardized scoring systems for severity of illness (*30*) and from careful analyses of outcome data in relation to drug exposure. Recent developments in culture sampling, such as nasal catheterization in patients with bacterial sinusitis (*31*), may allow serial observations of antimicrobial drug effects over time and avoid the bias introduced by solely evaluating treatment failures. In principle, continuous sampling of urine in patients with urinary tract infections and the analogous monitoring of drug concentrations and outcomes in middle-ear and lower respiratory infections may also be possible. However, the invasive nature of such studies may preclude a mandatory role in routine antibacterial drug development and licensing.

In April 2004, a workshop cosponsored by the US Food and Drug Administration (FDA) concluded that PK/PD research is useful in dose selection, that modeling and simulation tools may be used to quantitatively predict microbiologic outcomes and account for PK variability, and that PK/PD relationships generated from nonclinical studies should be confirmed in well-designed clinical studies (http://www.fda.gov/cder/drug/antimicrobial/FDA_IDSA_ISAP_Presentations.htm). As a tool for both regulatory agencies and the pharmaceutical industry, PK/PD studies can provide critical information to help 1) guide the development of optimal dosing schedules for clinical trials and minimize the selection of resistant bacteria during routine clinical use; 2) translate evolving MIC susceptibility data into dosing and treatment recommendations in the absence of data on the clinical effect of resistance; and 3) identify areas where resistance patterns most threaten the efficacy of existing therapies and help identify priorities for new drug development.

## Regulatory Responses to Resistance

Regulators are primarily concerned with licensing new drugs by verifying their safety, efficacy, and quality. Regulators also have roles that relate to the long-term safety of established agents by responding to postlaunch data. In some countries, licensing authorities regulate the fiscal effect of new therapies, while other countries rely on market forces or employ other agencies to assess cost-effectiveness. In addition, regulators share some responsibility for the sustainability of licensed agents through refinement of indications and recommendations.

The activities of 4 regulatory agencies were discussed during the International Forum on Antibiotic Resistance (IFAR) 2003 colloquium, namely those of Australia, the United States, France, and the European Union (EU) (Table). These activities represent a range of approaches to antimicrobial drug regulation but do not represent a comprehensive sample.

**Table:** Measures taken by selected regulatory agencies before and after licensing to assess and control antimicrobial drug resistance*

Measure	Australia	France	European Commission	United States
Primary drug registration body	Therapeutic Goods Administration, Australian Drug Evaluation Committee	AFSSAPS (French health products safety agency), Commission for Marketing Authorization	European Medicines Evaluation Agency†	Food and Drug Administration‡
Drug resistance advisory resource	EAGAR	GTA, CA-SFM, ONERBA	EARSS	AIDAC
Licensing				
	Use of supportive PK/PD data	Yes	Yes	Yes	Yes
	Risk assessment	Yes	Yes	Yes	Yes
After licensing				
	Prescription-only status	Yes	Yes	NA	Yes
	Community drug subsidy restrictions	Yes	No	NA	No
	Participation in education (e.g., guidelines)	Yes	Yes	No	Yes
	Directives on drug use	Yes	Yes	No	No
	Indication review based on resistance	No§	Yes	Yes	Yes
	SPC update/harmonization	Yes¶	Yes	Yes	Yes

*AFSSAPS, Agence Française de Sécurité Sanitaire des Produits de Santé; EAGAR, Expert Advisory Group on Antimicrobial Resistance; GTA, Groupe de Travail Anti-infectieux; CA-SFM, Comité de l'Antibiogramme de la Société Française de Microbiologie; ONERBA, Observatoire National de l'Epidémiologie de la Résistance Bactérienne aux Antibiotiques; EARSS, European Antimicrobial Resistance Surveillance System; AIDAC, Anti-Infective Drugs Advisory Committee; PK/PD, pharmacokinetic/pharmacodynamic; NA, not applicable; SPC, summary of product characteristics. †Scientific opinions are prepared by committees for human medicinal products, veterinary products, and orphan products. ‡Wider issues involving drug resistance, such as surveillance and appropriate use, are the purview of a number of United States agencies, including the Food and Drug Administration but also the Centers for Disease Control and Prevention, the National Institutes of Health, and other agencies partnering in the United States Public Health Action Plan to Combat Antimicrobial Resistance initiated in 2001 (*2*). §Only possible for animal antimicrobial drugs. ¶Agreement has been made to update SPCs every 5 years with Australian surveillance data. However, a mechanism for collecting these data has yet to be agreed upon.

In Australia, registration of drugs for human use is undertaken by the Therapeutic Goods Administration, which is supported by the Australian Drug Evaluation Committee. Both groups are advised by the Expert Advisory Group on Antimicrobial Resistance. In the United States, FDA is responsible for reviewing the safety and efficacy of antimicrobial drugs. When appropriate, FDA solicits input from its Anti-infective Drugs Advisory Committee. The wider issues involving antimicrobial drug resistance, such as surveillance and appropriate use, are the purview of a number of agencies, including FDA, the Centers for Disease Control and Prevention (CDC), and the National Institutes of Health (NIH) (*2*).

Antimicrobial drug licensing at the French Health Products Safety Agency involves an external, multidisciplinary antiinfectives working group, the Groupe de Travail Anti-infectieux. Drug licensing at the EU level is performed either through a centralized procedure mediated by the European Medicines Evaluation Agency (EMEA) or a decentralized procedure based on mutual recognition among member states after the initial step of a national market authorization in a state. Information on drugs registered at the EU level is described in a common European summary of product characteristics document. The EU Committee for Human Medicinal Products guides industry in developing medicines and identifies key information required for licensing (*32*). FDA supplies similar guidance to drug developers (http://www.fda.gov/cder/guidance/index.htm), and guidance on developing agents to treat resistant pathogens is under development.

EMEA (*33*) and FDA encourage drug developers to submit supportive PK/PD data. For example, if in vitro and PK/PD studies show that a drug has similar activity against strains that are susceptible or resistant to existing agents, clinical data against susceptible strains may support efficacy against resistant strains (although clinical data against resistant strains will ultimately be necessary).

### Scheduling and Subsidy Restriction

Most developed countries categorize antimicrobial drugs within a "prescription-only" schedule, thereby preventing over-the-counter sales and giving physicians and other healthcare professionals responsibility for their distribution. Restrictions on the subsidization of prescription costs paid by patients in the community may be a means of controlling state-funded drug use. In Australia, prescriptions for certain antimicrobial drugs are not subsidized unless the prescriber gains approval for their use (in specific indications) from the central Pharmaceutical Benefits Scheme. This system has resulted in low levels of fluoroquinolone use and resistance (*34**,**35*). However, differential subsidy levels may simply shift drug use toward cheaper agents, and consequently, subsidy restriction may be more useful in controlling the types of drugs prescribed, rather than the gross quantity. In the United States, where cost controls are not used, a decrease in prescribing has been accompanied by an increase in the use of newer, more expensive, and broad-spectrum agents (*36*). However, this increase may be the result of industry marketing forces rather than the lack of subsidy restrictions.

### Prescribing Directives and Guidance

Regulators may issue directives to prescribers regarding antimicrobial drug use. However, these must be carefully planned and implemented to avoid disadvantageous effects on prescribing behavior (*37*). FDA issues licensed indications and can create mandatory regulatory policies for certain drugs. It also oversees the content of package inserts and advertisements. However, as in other countries, prescribing practices are at the discretion of the individual clinician.

Regulatory authorities may be involved in educational initiatives to improve antimicrobial drug use. In France, official guidelines on drug use underpin regulation, pharmaceutical promotion, and education. A recent national plan to promote judicious use involved amending antimicrobial drug summaries of product characteristics, as well as amending treatment guidelines and the provision of free streptococcal tests and information for patients and parents (B. Schlemmer, pers. comm.). In the United States, FDA and CDC have partnered on the Get Smart program (http://www.cdc.gov/getsmart), aimed at fostering appropriate antimicrobial drug use.

### Prescribing Information

The usefulness of resistance data within current prescribing information labels may be questioned, given the largely empiric nature of community antimicrobial drug prescribing. FDA has recognized the need to inform clinicians about resistance issues for empirically treated diseases and has designated several drugs, for which adequate clinical data exist, as safe and effective in the treatment of community-acquired pneumonia caused by multidrug-resistant *S. pneumoniae*. Updating labeling is a substantial undertaking. In 2003, labels for 669 drugs had to be changed when FDA amended labeling requirements for antimicrobial drugs (*38*).

In Europe, international disharmony remains in the summaries of product characteristics for older drugs. Efforts to update and harmonize these will require cooperation between EMEA, national regulatory bodies, and the pharmaceutical industry. Experience from Australia, where the registration system for human antimicrobial drugs has been revised to incorporate resistance risk assessment, suggests that this process will be challenging. As generic manufacturers have no responsibility to provide resistance data for their products, healthcare systems may have to provide resources to collect these data.

### Indication Review

Indication review is the process by which regulatory authorities reassess the licensed indications of a drug in light of new data. In some countries (e.g., Australia) indication review may only be performed on the basis of drug safety. In others, it may in principle be performed on resistance grounds. Any decision to change a drug's license should be based on robust clinical evidence of a public hazard. In vitro surveillance data may be insufficient in isolation, as previously discussed. Moreover, uncertainty exists about the threshold resistance prevalence at which indications should be withdrawn.

### Incentives to Antimicrobial Drug Development

In principle, the current decline in drug development could be reversed by a number of means. Substantial costs are incurred by the late-stage failure of developmental candidates. Costs may be reduced by efficiently identifying drugs that are more likely to be effective, allowing earlier decisions on development cessation, which is the focus of the FDA Critical Path Initiative (http://www.fda.gov/oc/initiatives/criticalpath/). Public-industry risk sharing could also be considered for phase III trial funding. Detailed PK/PD investigations could potentially reduce the number of phase I/II studies required (*33*) and facilitate dose selection for phase III trials. Other possible approaches include the use of data in 1 indication to support a license application in another (providing the spectrum of causative pathogens, PK/PD factors, and infection severity is sufficiently similar). Regulatory authorities have offered fast-track designation and priority review for narrow-spectrum antimicrobial drugs and agents active against multidrug-resistant organisms. However, FDA grants priority reviews on the basis of results of clinical trials with a drug, not on in vitro spectrum alone.

Recently, fruitful collaborations have taken place between regulatory agencies, healthcare systems, academia, and industry. FDA has consulted with representatives of the pharmaceutical industry and IDSA and has identified pathogens of primary public health importance (http://www.fda.gov/ohrms/dockets/ac/03/slides/3931S2_03_Powers_files/frame.htm). IDSA has held preliminary discussions with NIH to explore ways in which trial funding could be shared between public bodies and industry. However, considerable political, logistic, and financial challenges must be overcome if public-private partnership models are to be applied.

Financial incentives could be provided to industry by waiving or reducing the new drug application fee, by extending or renewing patents for antimicrobial drugs of public health priority, or by granting orphan drug status for treatments for serious but rare diseases. "Wild card" measures are an alternative approach, whereby a company can choose which drug in its portfolio is granted exclusivity or patent extension. Considering government contracts with industry for specific agents or guaranteeing markets for niche drugs may have value. More widely, opportunities may exist to reconsider drug pricing structures and tax incentives related to antimicrobial drug revenues. Because regulatory bodies can only act within existing legislation, legislative changes may be required to provide economic incentives to industry.

The provision of such incentives should be dependent on responsible marketing and sales activities by pharmaceutical companies. In the United States, the Department of Health and Human Services Office of the Inspector General has developed guidelines for marketing activities that have been adopted by many companies (http://oig.hhs.gov/authorities/docs/03/050503FRCPGPharmac.pdf).

The development of narrow-spectrum antimicrobial drugs or adjunctive agents that target specific resistance mechanisms will not be viable without effective, low-cost diagnostic methods available at the point of prescribing. Thus, incentives must also be considered for the development and clinical adoption of diagnostic technologies.

## Conclusions

Regulatory authorities must balance the requirements for safe and effective medicines, and the need for new antimicrobial drugs effective against resistant pathogens, with the technologic and commercial realities of drug development. We do not know whether the development of new antimicrobial drugs will keep pace with the emergence of resistant pathogens. This uncertainty highlights a need to identify gaps in available drugs and for governments to devise innovative regulatory and legislative measures to stimulate the development of new agents and diagnostic technologies.

PK/PD models that integrate preclinical and clinical data offer a promising approach to defining optimal drug doses for phase III clinical trials. PK/PD data may also help define the determinants of resistance selection, quantify the clinical effect of resistance, and identify where resistance patterns most threaten the efficacy of existing therapies and where priorities for drug development lie. However, further clinical research is required to correlate microbiologic outcomes based on PK/PD data and clinical outcomes in patients. These trials should exploit recent advances in novel endpoints, sampling techniques, and PK modeling. Potentially, these data may be used in conjunction with outcomes research in determining susceptibility breakpoints for clinical purposes.

Initiatives in Europe and the United States indicate a welcome trend toward greater consultation and collaboration between regulatory authorities, the pharmaceutical industry, and knowledgeable professionals. The role played by regulatory authorities in controlling drug use varies by country. In this context, efforts to improve regulatory measures would benefit from greater international dialogue.
